# Posterior pilon fracture treated by opening the fibula fracture gap

**DOI:** 10.1186/s13018-022-03106-4

**Published:** 2022-04-07

**Authors:** Zhuang Jiang, Chen Zhang, Jia-Jun Qin, Guo-Dong Wang, Hua-Song Wang

**Affiliations:** 1 Orthopaedic Department, General Hospital of Central Theater Command of PLA, #627 Wuluo Road, Wuchang District, Wuhan, 430070 China; 2 Clinical Medicine, Wuhan University of Science and Technology, #2, West Huangjiahu Road, Hongshan District, Wuhan, 430081 China

**Keywords:** Posterior pilon fractures, Trimalleolar ankle fractures, Ankle, Syndesmotic injury, Fibular fracture

## Abstract

**Background:**

Posterior pilon fracture is a relatively common clinical fracture involving the posterior articular surface of the distal tibia. Currently, this form of fracture is receiving increasing attention. The surgical approach and technique for the treatment of posterior pilon fractures are still controversial. The purpose of this retrospective study was to compare the clinical and imaging outcomes of pilon fractures after treatment with the open fibula fracture line (OFFL) surgical technique versus the traditional posterolateral approach (TPL).

**Methods:**

A retrospective analysis of patients with posterior pilon fractures treated using the open fibula fracture line technique and the traditional posterolateral approach between January 2015 and March 2020. Thirty-one cases were included in the open fibula fracture line technique group and twenty-eight cases were included in the traditional posterolateral approach group. We used the Burwell-Charnley scale to assess the effectiveness of surgical repositioning. The clinical outcomes were evaluated using American Orthopaedic Foot & Ankle Society ankle-hind foot score (AOFAS) and visual analog score (VAS).

**Results:**

The overall anatomic reduction rate was slightly better in the open fibula fracture line group than in the conventional posterolateral group (81% vs. 71%, *p* = 0.406), but there was no statistically significant difference between the two groups. There were no statistically significant differences between the two groups in terms of fracture healing time and time to full weight bearing (*p* > 0.05). At the final follow-up, the AOFAS functional score of the open fibula fracture line group was statistically superior to that of the conventional posterolateral group (*p* < 0.05). However, there was no statistical difference between the two groups in VAS pain scores at rest, during activity, and under weight bearing (*p* > 0.05).

**Conclusion:**

The trans-fibular fracture approach provides a better surgical option for specific types of posterior pilon fractures with a high rate of anatomic repositioning and a good near-term outcome.

*Trial registration*: Retrospective registration.

## Introduction

Pilon fracture is a compression fracture of the entire distal tibial articular surface caused by high energy vertical violence. In contrast, posterior pilon fractures are the result of a combination of vertical and rotational violence and involve only the posterior articular surface of the distal tibia [1, 2]. The energy tends to be less than that of pilon fracture, and the soft tissue condition is superior to that of pilon fracture. Posterior pilon fractures are produced by vertically and rotational stresses on the foot in plantar flexion and are characterized by major fracture mass on the posterior side, a coronal fracture line, and the possible inclusion of a die-punch bone mass [3]. The prognosis is not as good as for posterior ankle fractures due to the large area of involvement of the posterior articular surface of the distal tibia [4, 5].

The interest of foot and ankle scholars in posterior pilon fractures has grown since the concept of posterior pilon fractures was introduced. Our team believes that posterior pilon fractures involve the posterior articular surface of the distal tibia and that anatomic repositioning is mandatory. Currently, there are many surgical approaches for posterior pilon fractures, such as the posterolateral approach, the posterior-medial approach, or the modified posterior-medial approach [2, 6–11]. However, the best treatment for posterior pilon fracture is a matter of opinion.

The posterior pilon fracture line is mostly coronal, with step production, longitudinal displacement of the articular surface, and easy entrapment of articular cartilage and soft tissue. Posterior pilon fractures should be dissected and repositioned as much as possible to reduce the occurrence of traumatic arthritis. The main advantage of the posterolateral approach is that a single incision can treat both the posterior ankle and fibula, but it does not adequately expose the tibial fracture end and articular surface, and poor repositioning is often encountered. If adequate exposure is required, it is necessary to turn the posterior bone block, which is more disruptive to the soft tissues. Therefore, we considered whether posterior pilon fractures with combined fibular fractures could be treated by opening the fibular fracture line through a posterior lateral approach to look directly at the distal tibial articular surface, which would reduce irritation to the posterior soft tissues and neurovascular, etc. However, the fibular fracture line and the tibial fracture line may not be in the same plane, which is not applicable for high fibular fractures, or may require distal osteotomy. The same approach is used for combined posterior ankle fractures, especially the posterior rotation type.

The purpose of this study was to retrospectively examine the clinical outcomes of the open fibula fracture line technique compared with the traditional posterolateral approach for posterior pilon fractures. In the article, we will discuss the surgical technique, advantages and limitations of this approach.

## Material and methods

### Study design and patient population

This study was a retrospective study. The study was approved by the Ethics Committee of General Hospital of Central Theater Command and was in accordance with the Helsinki Declaration.

Sixty-one patients met the inclusion criteria and two patients were lost to follow-up. A total of 59 patients were included in this study, 31 in the open fibula fracture line group and 28 in the traditional posterolateral group. Patients who underwent surgery using OFFL or TPL for the treatment of posterior pilon fractures between January 2015 and March 2020 were included. Our inclusion criteria were (1) Preoperative patients were perfected with X-ray and CT 3D reconstruction examinations. (2) The diagnosis of posterior pilon fracture is clear. (3) Combined fibula fracture and fibula fracture line around the ankle joint. (4) Closed fracture and normal ankle function before the injury. Our exclusion criteria were (1) Patients who have lost visits. (2) No combined fibula fracture. (3) Combined fibula fracture, but the fibula fracture line is not near the ankle joint. (4) Those who cannot tolerate surgery.

### Surgical technique

**Fig. 1:**
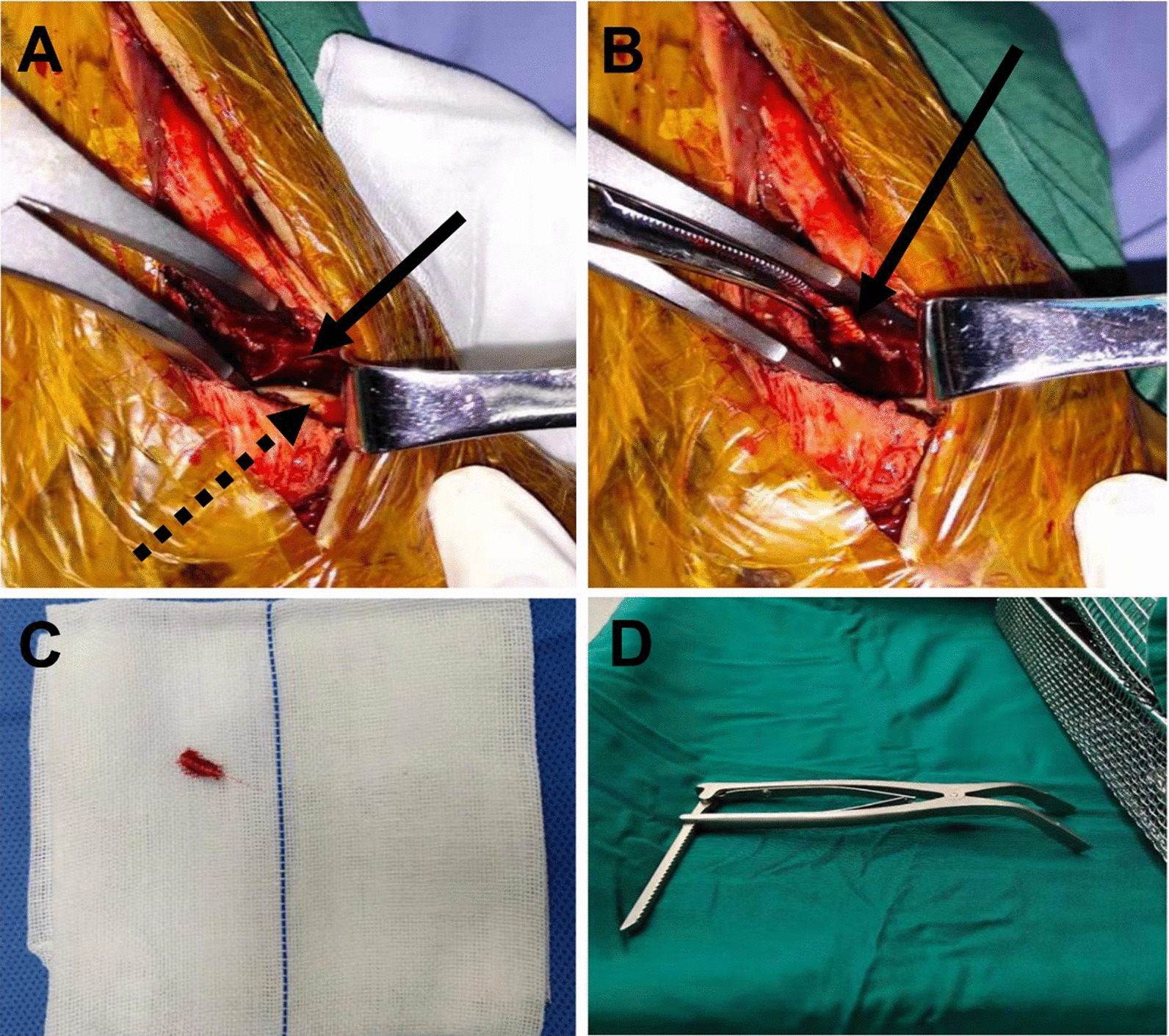
Surgical procedure to open the fibula fracture line. **A** The distal tibial articular surface and the talar articular surface can be clearly visualized by opening the fractured end of the fibula with a spreader. The solid black line is the distal tibial articular surface and the dotted black line is the talar articular surface. **B** A small fragment of bone inserted between the fracture ends. The solid black line is the small bone mass. **C** Intraoperative removal of small fragments that interfere with fracture reduction. **D** Physical view of the intraoperative spreader

In the open fibula fracture line group (OFFL). General anesthesia or continuous epidural anesthesia was administered. Patients were uniformly placed in the floating position. A tourniquet is often applied in the upper 1/3 of the thigh. A longitudinal incision is made along the posterior lateral border of the fibula, with the distal end of the incision arcing slightly forward. The fibular fracture line is revealed on the anterior aspect of the longus and brevis peroneus muscles, and then, the posterior tibial bone mass is revealed between the longus and brevis peroneus muscles and the long and flexor thumb muscles. It is important to note that the sural nerve and small saphenous vein need not be deliberately exposed during superficial separation. Instead, it is protected by soft tissue and pulled posteriorly. The fibula longus and brevis muscles and the flexors hallucis longus are separated in the deep layer. At this time, attention should be paid to the protection of the fibula artery. At this point, care needs to be taken to protect the fibular artery. After exposure is complete, the fracture end of the fibula is cleaned and the fibula fracture line is propped open with a spreader to reveal the deep posterior pilon fracture articular surface (Fig. 1A). If a suitable spreader is not available, the fracture end can be opened with a K-wire spreader, the principle of which is the same: A thick K-wire is drilled into each end of the fibula fracture, and then, a K-wire spreader is applied to open the fracture end. The tibial articular surface is then pried open under direct vision with a periosteal stripper to clean the embedded articular cartilage and soft tissues, restore the flatness of the articular surface, and correct the longitudinal displacement of the bone mass (Fig. 1B). In theory, the die-punch bone with the articular surface should be repositioned, but if the bone is too small to be fixed, we will simply remove it (Fig. 1C). Avoid interfering with the postoperative joint motion. The posterior fracture is then first temporarily fixed using a K-wire. At this point, the distal tibial articular surface is observed directly in the fibular space to see if it returns to level. Of course, fluoroscopy can also be performed to assess the smoothness of the joint surfaces. After confirming that the distal tibial articular surface is flat, the screws or plates are selected for fixation, depending on the situation. After the posterior bone block was fixed, the distal tibial articular surface was again observed through the fibular space to see if any screws entered the joint cavity. The fibula fracture was then fixed with an anatomic plate of the fibula. Depending on the medial ankle fracture, a small medial incision is made for the repositioning and fixation of the medial ankle fracture. The incision is finally closed in layers and the incision is aseptically dressed without the need for drainage placement. Rinse; suture; dressing, loosening of tourniquet, end of procedure.

In the traditional posterolateral group (TPL), preoperative preparation was as before. A standard posterolateral incision is used. The long and short fibular muscles and the long flexor of the thumb are exposed. The posterior aspect of the ankle is revealed by accessing through the muscle gap to reveal the posterior pilon fracture. Care is taken to protect the common peroneal nerve and the lesser saphenous vein during exposure. The posterior bone block is turned over by means of an open book fashion to reveal and treat the embedded articular bone block or soft tissue, and the K-wire is temporarily fixed. Then, depending on the degree of fracture comminution, appropriate size plates and screws are selected for fixation. Fractures of the fibula and medial ankle are treated as above.

### Postoperative management

All patients in both groups received the same postoperative management and follow-up protocol. The intraoperative situation determines whether to continue postoperative plaster immobilization. Postoperative plaster fixation was taken in 16 cases in the open fibula fracture line group and in 18 cases in the traditional posterolateral group. All of them had their cast removed after three weeks for functional exercise. The rest of the patients gradually started active and passive exercises on the second postoperative day.

### Follow-up

The patients were reviewed and followed up immediately after surgery, 1 month after surgery, 2 months after surgery, and 3 months after surgery, respectively. Thereafter, patients were followed up at 3-month intervals.

### Clinical and radiographic assessment

The time of surgery and the number of fluoroscopic were recorded. Postoperatively, the Burwell–Charnley scoring system was adopted for the assessment of the repositioning effect. The time to fracture healing and the time to be able to fully bear weight were observed during the follow-up. Complications were recorded at follow-up. At the last follow-up, the American Orthopedic Foot and Ankle Score (AOFAS) [12] and visual analog score pain score (VAS) were taken to evaluate the clinical outcome.

### Statistical analysis

Statistical analyses were performed with SPSS 25.0 software. Quantitative data were expressed as the mean ± standard deviation. Independent samples t test was used for conformity to normal distribution, and nonparametric test was used for non-conformity. Qualitative data were shown as the frequency (%). The chi-square test was used to compare the qualitative data (χ^2^).

## Results

### Patients demographic data

A total of 59 patients met the inclusion criteria, with 31 in the open fibula fracture line group and 28 in the traditional posterolateral group. The demographic information about the included patients is shown in Table 1.

**Table 1:** Demographic data of patients

Factor	OFFL (*n* = 31)	TPL (*n* = 28)	*t* test	*p* value
Age at injury (years)	48.3 ± 13.2	45.0 ± 12.7	*t* = 0.970	0.336
Male sex (*n*)	14 (45%)	14 (50%)	*χ*^2^ = 0.138	0.710
Causes of injury (*n*)			*χ*^2^ = 0.157	0.925
Falls on sloping roads	13 (42%)	13 (46%)		
Falls on flat roads	9 (29%)	8 (29%)		
Traffic accidents	9 (29%)	7 (25%)		
Combined ankle subluxation	13 (42%)	9 (32%)	*χ*^2^ = 0.603	0.437
Combined die-punch fragments	16 (52%)	14 (50%)	*χ*^2^ = 0.015	0.902
Combined fractures of the medial malleolus	15(48%)	12(43%)	*χ*^2^ = 0.181	0.670

Open fibula fracture line group (OFFL); traditional posterolateral group (TPL)

### Clinical and radiographic outcomes

**Fig.2:**
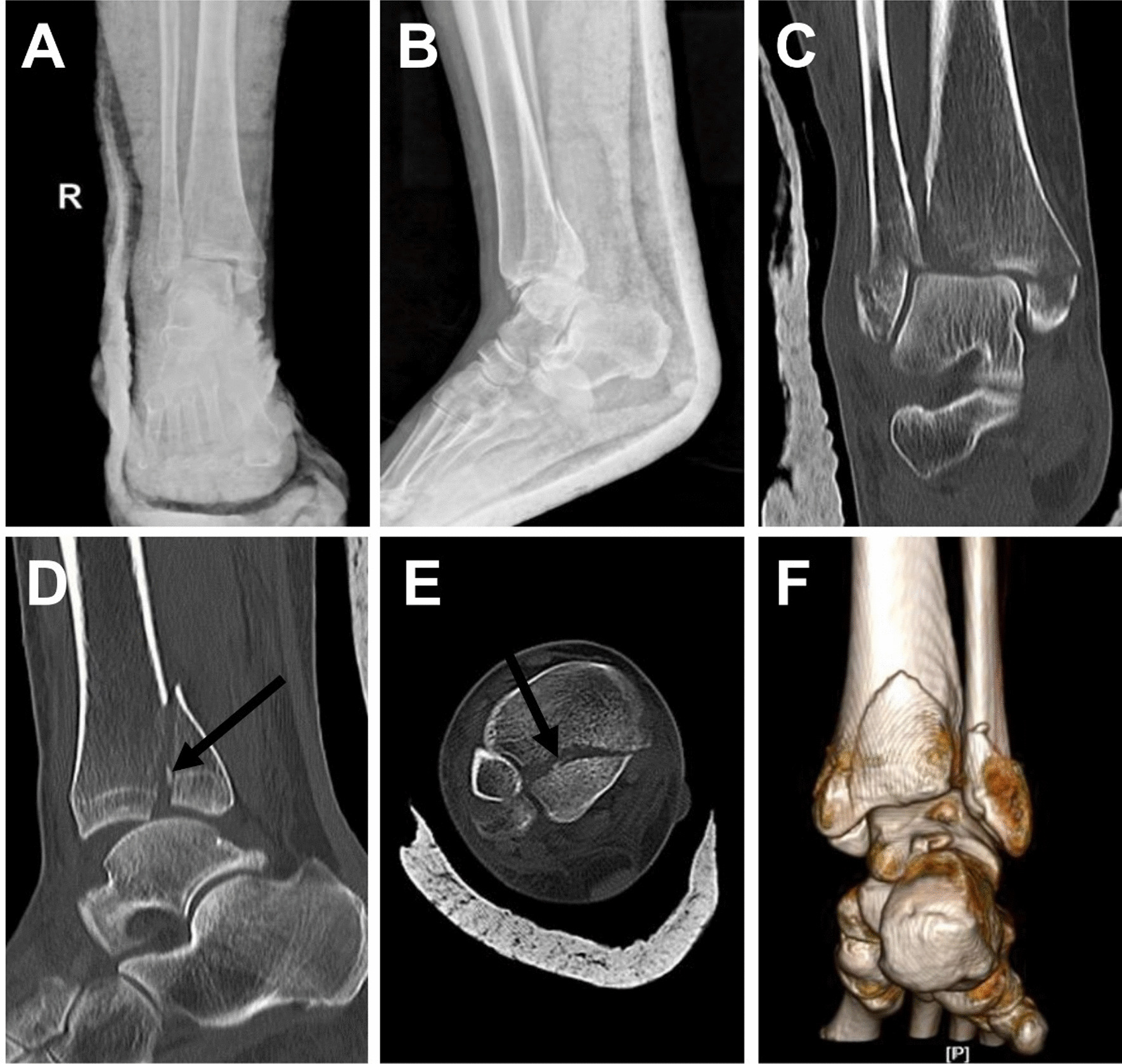
Preoperative X-ray and CT 3D reconstruction of the patient. **A**, **B** X-ray in anterior–posterior and right-left positions. **C** CT coronal plane. **D** The sagittal plane shows a subluxation of the talus with a clear step sign. **D**, **E** The small embedded bone can be clearly seen in the sagittal and transverse views, as indicated by the solid arrows. **F** Representative image of CT 3D reconstruction

**Fig. 3:**
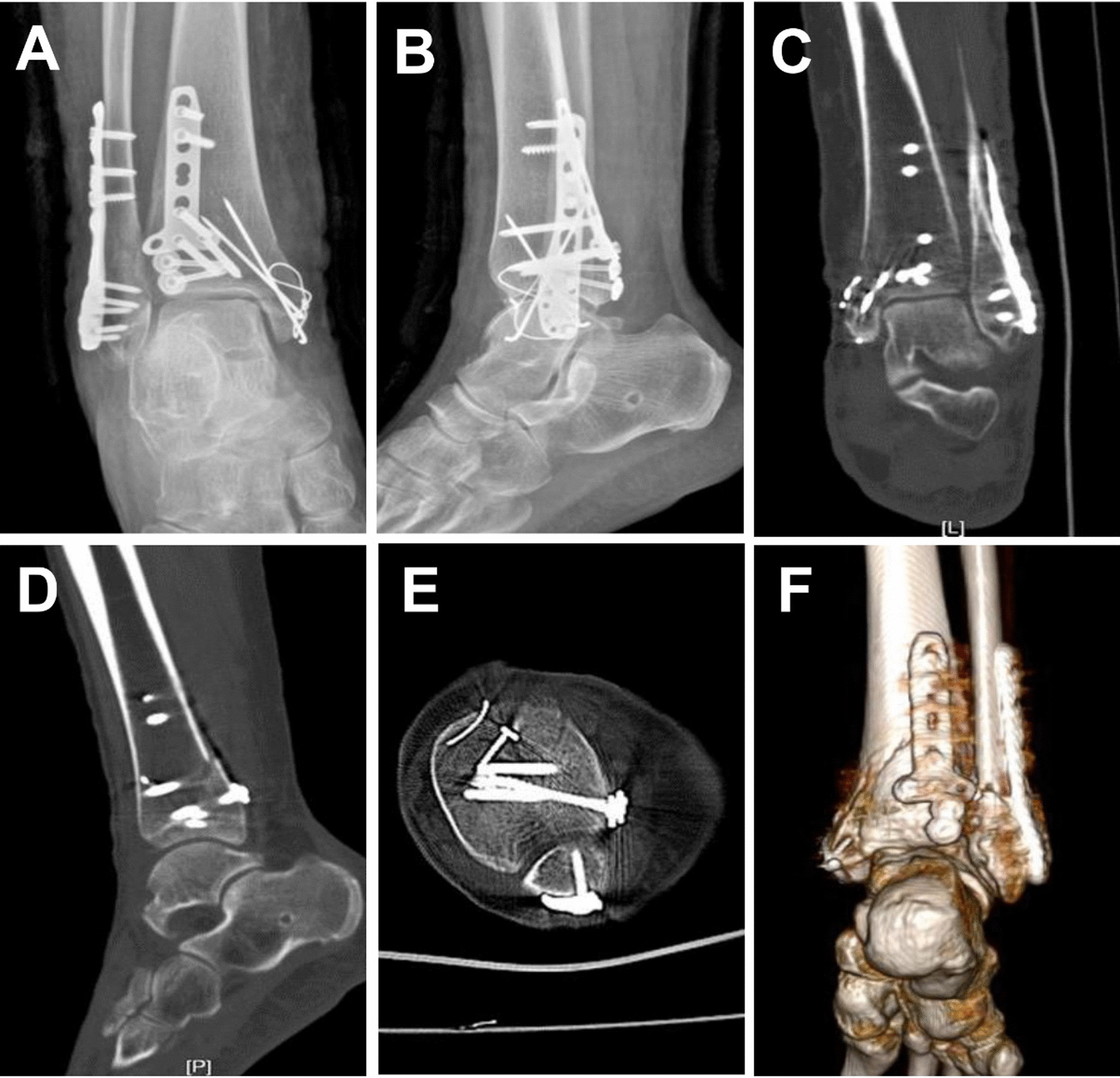
The patient’s postoperative X-ray and CT 3D reconstruction showed anatomic reduction in the fracture. **A**, **B** X-ray in anterior–posterior and right-left positions. **C** CT coronal plane. **D** The sagittal subluxation and step sign were corrected. **E** CT transverse views. **F** Representative image of CT 3D reconstruction

Fracture healing time (2.7 months ± 0.7 vs. 3.0 months ± 1.0) and time to full weight bearing (3.4 months ± 0.5 vs. 3.7 months ± 0.8) were not significantly different in the two groups. Postoperative assessment was performed using the Burwell–Charnley score. The overall anatomic repositioning rate was better in the open fibula fracture line group than in the conventional posterolateral group (81% vs. 71%), but not statistically significant. At the final follow-up, there was no significant difference between the two groups in VAS pain scores, (rest 0.5 ± 0.6 vs. 0.6 ± 0.5) (activity 0.9 ± 0.8 vs. 1.1 ± 0.8) (weight-bearing walking 1.5 ± 0.9 vs. 1.6 ± 0.9). However, the open fibula fracture line group was significantly better than the traditional posterolateral group (86.6 ± 7.1 vs. 82.7 ± 6.9, *p* = 0.037) for the AOFAS score. (Table 2). Preoperative imaging data on typical cases are shown in Fig. 2. Postoperative imaging data on typical cases are shown in Fig. 3.

**Table 2:** Clinical and radiographic outcomes

Factor	OFFL (*n* = 31)	TPL (*n* = 28)	*t* test	*p* value
Fracture healing time (month)	2.7 ± 0.7	3.0 ± 1.0	*t* = − 1.498	0.140
Full weight-bearing time (month)	3.4 ± 0.5	3.7 ± 0.8	*t* = − 1.455	0.151
Anatomic repositioning (*n*)	25 (81%)	20 (71%)	*χ*^2^ = 0.691	0.406
VAS (rest)	0.5 ± 0.6	0.6 ± 0.5	*t* = − 1.009	0.313
VAS (activity)	0.9 ± 0.8	1.1 ± 0.8	*t* = − 1.224	0.221
VAS (weight-bearing walking)	1.5 ± 0.9	1.6 ± 0.9	*t* = − 0.271	0.786
AOFAS	86.6 ± 7.1	82.7 ± 6.9	*t* = 2.133	**0.037**

Bold indicates statistically significant

*OFFL* open fibula fracture line group, *TPL* traditional posterolateral group, *AOFAS* American Orthopedic Foot and Ankle Score, *VAS* visual analog score pain score

### Complications

Surgery-related complications include impaired wound healing, bone discontinuity, vascular nerve damage, and pain. However, there was no statistically significant difference in complications between the two groups, 16% and 25%, respectively (*p* = 0.398) (Table 3).

**Table 3:** Complications

Factor	OFFL (*n* = 31)	TPL (*n* = 28)	*t* test	*p* value
Total number of patients with complications (*n*)	5 (16%)	7 (25%)	*χ*^2^ = 0.715	0.398
Vascular nerve injuries (*n*)	2 (6%)	3 (11%)		
Soft tissue complications (*n*)	2 (6%)	2 (7%)		
Postoperative pain (*n*)	1 (3%)	2 (7%)		

All variables were reported in terms of counted cases and relevant percentages and compared with the *χ*^2^ test

### Surgical time and Fluoroscopic times

The operative time for the open fibula fracture line group and the conventional posterolateral group was 109.6 ± 7.7 min and 115.7 ± 6.0 min, respectively, with statistically significant differences (*p* = 0.001). The number of intraoperative fluoroscopic views was 9.0 ± 1.3 and 9.5 ± 1.5 in the two groups, respectively, with no statistically significant difference (*p* = 0.207) (Table 4).

**Table 4:** Surgical time and Fluoroscopic times

Factor	OFFL (*n* = 31)	TPL (*n* = 28)	*t* test	*p* value
Surgical time (min)	109.6 ± 7.7	115.7 ± 6.0	*t* = − 3.391	**0.001**
Fluoroscopic times	9.0 ± 1.3	9.5 ± 1.5	*t* = − 1.261	0.207

Mean ± standard deviations for the two groups, and relevant comparison performed by means of *t* test

Bold indicates statistically significant

## Discussion

The posterior pilon fracture is a special type of fracture. In clinical work, posterior pilon fractures are not uncommon. The concept of posterior pilon fracture was born relatively late [13]. Before the posterior pilon fracture was fully recognized, it was described as a “trimalleolar pilon fracture” [14]. Topliss retrospectively analyzed 108 pilon fractures based on CT imaging and reported that the incidence of posterior pilon fractures accounted for 5.6% of them [4]. While Chen retrospectively analyzed 157 cases of triple ankle fractures, posterior pilon fractures accounted for approximately 6.4% [15]. Posterior pilon fracture is not like a traditional trimalleolar fracture (low energy rotational stress) or a classic pilon fracture (high energy vertical violence). However, posterior pilon fractures have both of these characteristics. It is usually the result of vertical and rotational stresses when the ankle is in plantarflexion position. A coronal fracture line is formed, often accompanied by compression of the posterior articular surface of the distal tibia and persistent posterior talar subluxation [15, 16]. In contrast to classic posterior ankle fractures, in posterior pilon fractures, the fracture is wedge-shaped, may have two major bone masses (posterolateral and posteromedial), involves a large area of the posterior articular surface of the distal tibia [17], and the fracture line may involve the posterior malleolus of the medial ankle. It may be accompanied by a die-punch bone mass. Posterior pilon fractures are relatively unrecognizable on conventional radiographs. The appearance of “double contour” or “double joint line sign” on the anteroposterior X-ray should be highly suspected of posterior pilon fracture [5, 14, 18, 19]. For further evaluation, 3D CT reconstruction is recommended to better understand the fracture pattern and to facilitate appropriate surgical planning [3, 13, 14, 20–23]. Currently, there is no classification that fully summarizes the characteristics of posterior pilon fractures. The ideal fracture classification will suggest the mechanism of injury, guide treatment, and predict prognosis. A representative type is the Klammer type [2]. Klammer classified the posterior pilon into three types based on the primary site of the posterior bone fragment to guide the strategy of surgical treatment.

Numerous surgical procedures are currently available to treat posterior pilon fractures, but posterior posterolateral is the most commonly used. This is because it allows one incision to fix the posterior and lateral ankle [2, 6–11]. The current surgical challenge is to adequately visualize the distal tibial fracture break and to deal with small bone fragments embedded in the fracture, such as die-punch bone fragments, cartilage pieces and soft tissue. Therefore, the rate of anatomic resetting in previous studies was not too high [2, 11]. Klammer uses an “open book fashion” technique to open the posterior fracture to reveal the tibial articular surface [2]. There is no doubt that this will destroy more soft tissue on the posterior side. In our traditional posterolateral group, the distal tibial articular surface is also revealed in an “open book fashion.” In the case of this retrospective controlled study, the TPL group will have a longer operating time and more posterior lateral soft tissue damage.

Previous papers have reported that most posterior pilon fractures are combined with fibular fractures (external ankle fractures) and tend to be posterior-superior to anterior-inferior oblique fracture lines rather than spiral fractures [5, 16]. Our team also found this feature in our clinical work and most of the fibula fracture lines were near the ankle joint. So our team proposed to use the fibular fracture gap to treat posterior pilon fractures. The results were exciting, with a high rate of anatomic repositioning and significant correction of the distal tibial articular surface. Embedded articular cartilage and soft tissue at the fractured end increase the risk of poor fracture repositioning and fracture nonunion, and complete removal of the embedded tissue under direct vision improves the anatomic repositioning rate and reduces the risk of fracture nonunion. Also, small compression and overturning of the articular surface of the distal tibia can be better corrected (using the talus as a template for splicing the articular surface). The results of this study showed that the anatomic repositioning rate in the OFFL group was better than that in the TPL group (81% vs. 71%). The AOFAS score was significantly higher in the OFFL group than in the TPL group at the final follow-up (*p* = 0.037). Also using the traditional posterolateral approach, the anatomic repositioning rate was higher than the 53.8% of Klammer et al. [2] and the 73.9% of Gao et al. [11]. The AOFAS score was also higher than the 82 points of Klammer et al. [2] and the 82.3 points of Gao et al. [11].

Of course, our drawbacks are also obvious. This study had a relatively small sample size due to the strict indications. It is a retrospective study, not a prospective cohort study, and has some limitations. First of all, this method will never work for patients who do not have a combined fibula fracture. Secondly, although a combined fibula fracture with a high fracture line is still not possible, moreover, a combined medial incision is still required for fractures involving the medial ankle. Definitely, the current staging of posterior pilon fractures focuses on the size and location of the posterior fracture and the Die-punch fragment. It does not take into account the fracture of the fibula. Thus, this may lay the groundwork for a more comprehensive typology to be proposed later.

## Conclusion

In conclusion, the treatment of posterior pilon fractures remains challenging. We summarize the cases of these special posterior pilon fractures and propose a new protocol for the treatment of these special posterior pilon fractures. It provides an adequate visualization of the surgical field and has a satisfactory short-term outcome.

## Data Availability

The datasets for this study are available from the corresponding author on reasonable request.

## Abbreviations

OFFL: Open fibula fracture line group
TPL: Traditional posterolateral group
AOFAS: American Orthopedic Foot and Ankle Score
VAS: Visual analog score pain score
